# Formation of bacterial pilus-like nanofibres by designed minimalistic self-assembling peptides

**DOI:** 10.1038/ncomms13482

**Published:** 2016-11-17

**Authors:** Tom Guterman, Micha Kornreich, Avigail Stern, Lihi Adler-Abramovich, Danny Porath, Roy Beck, Linda J. W. Shimon, Ehud Gazit

**Affiliations:** 1 Department of Molecular Microbiology and Biotechnology, George S. Wise Faculty of Life Sciences, Tel Aviv University, Tel Aviv 6997801, Israel; 2 The Raymond and Beverly Sackler School of Physics and Astronomy, Tel Aviv University, Tel Aviv 69978, Israel; 3 Institute of Chemistry and The Center for Nanoscience and Nanotechnology, The Hebrew University of Jerusalem, Edmond J. Safra Campus, Jerusalem 91904, Israel; 4 Department of Oral Biology, The Goldschleger School of Dental Medicine, Sackler Faculty of Medicine, Tel Aviv University, Tel Aviv 69978, Israel; 5 Department of Chemical Research Support, Weizmann Institute of Science, Rehovot 76100, Israel; 6 Department of Materials Science and Engineering, Iby and Aladar Fleischman Faculty of Engineering, Tel Aviv University, Tel Aviv 6997801, Israel

## Abstract

Mimicking the multifunctional bacterial type IV pili (T4Ps) nanofibres provides an important avenue towards the development of new functional nanostructured biomaterials. Yet, the development of T4Ps-based applications is limited by the inability to form these nanofibres *in vitro* from their pilin monomers. Here, to overcome this limitation, we followed a reductionist approach and designed a self-assembling pilin-based 20-mer peptide, derived from the presumably bioelectronic pilin of *Geobacter sulfurreducens*. The designed 20-mer, which spans sequences from both the polymerization domain and the functionality region of the pilin, self-assembled into ordered nanofibres. Investigation of the 20-mer revealed that shorter sequences which correspond to the polymerization domain form a supramolecular β-sheet, contrary to their helical configuration in the native T4P core, due to alternative molecular recognition. In contrast, the sequence derived from the functionality region maintains a native-like, helical conformation. This study presents a new family of self-assembling peptides which form T4P-like nanostructures.

## Abstract (web summary)

The application of nanofibres formed from monomers of bacterial type IV pili *in vitro* has proven to be complex. Here, the authors have overcome this limitation by using a reductionist approach to design a self-assembling pilin-based 20-mer peptide capable of forming a supramolecular beta sheet.

## Graphical abstract

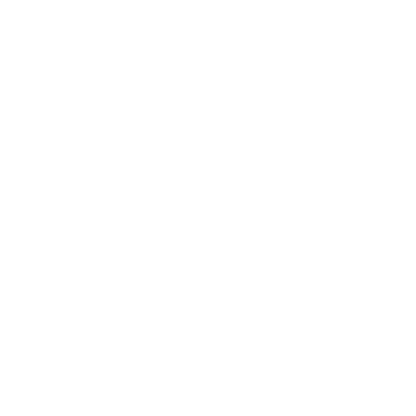

The study of protein self-assembly into ordered nanofibres in natural systems has given rise to a reductionist approach, whereby core peptide building blocks are derived from the parent protein. This approach has proven advantageous in establishing simpler *in vitro* model systems for studying the parent architecture. Importantly, this has also led to the development of new bio-inspired configurations that mimic the naturally occurring architectures while maintaining their functional properties, or even give rise to new functionalities. Prime examples of this approach are amyloid-like assemblies formed by peptide sequences that were identified as core recognition modules of amyloidogenic proteins^1^,^2^,^3^,^4^,^5^, superhelical and coiled coil fibres based on heptad repeats as inspired by intermediate filaments and keratin^6^,^7^,^8^,^9^, and collagen-like assemblies based on the characteristic collagen repeating sequence^10^,^11^. While such common protein polymers have been investigated and mimicked, similar study of the bacterial type IV pilus (T4P), an abundant class of polymeric nanofibres, has been very limited. This is due to the fact that intact pilins, the protein subunits of T4Ps, do not self-assemble *in vitro* into T4P-like structures^12^.

T4Ps are extracellular nanofibres that emerge from the cell surface of Gram-negative and Gram-positive bacteria and archaea^13^,^14^,^15^. These nanofibres are involved in diverse biological processes that include twitching motility, DNA transfer, biotic and abiotic surfaces adhesion, and extracellular electron transport^13^,^14^,^15^. Out of the different families of bacterial pili, T4Ps are the most widespread and appear in a plethora of species^16^,^17^. Structurally, T4Ps are supramolecular polymeric nanofibres of pilin^18^. The mature pilin generally comprises a long α-helix (α1) and a globular head domain that together form a ladle-like structure. The hydrophobic N-terminal segment of the α-helix (α1-N) is evolutionary conserved and is associated with pilus polymerization during biogenesis in the inner membrane, a highly concerted process executed by an intricate assembly complex^13^,^14^,^15^,^16^,^17^; this domain occupies the hydrophobic core of the mature T4P nanofibre^19^,^20^. The remaining head domain, which comprises a helical segment (α1-C), a β-sheet and several loops, is variable in sequence, subjected to post-translational modifications and exposed to its surroundings^19^,^20^. Accordingly, this region is responsible for the biological functionality of the T4P. Different T4Ps are capable of carrying out their specific biological functions owing to unique physicochemical properties. These include high mechanical flexibility^21^,^22^,^23^, charged exterior and high affinity towards various molecules and surfaces^24^,^25^ and electrical conductivity which is presumed to be intrinsic^26^,^27^,^28^. These properties present T4Ps as attractive systems to be mimicked for the development of functional nanostructured biomaterials.

The lack of success in inducing self-assembly of intact pilin subunits into T4P-like nanofibres *in vitro* poses a general limitation that hinders the development of applications based on these multifunctional structures. Yet, it was shown by Audette and co-workers that for the T4P of a *Pseudomonas aeruginosa* strain, this limitation can be overcome by engineering the pilin subunit^29^,^30^,^31^. This engineered pilin, lacking its hydrophobic α1-N polymerization region, spontaneously self-assembled into supramolecular nanofibres in the presence of hydrophobes, which presumably act as molecular surrogates for the missing hydrophobic α1-N region. Remarkably, the formed nanostructures were imparted with similar functionalities to those of the native pili. The very fact that engineering a pilin can lead to its *in vitro* self-assembly into T4P-like nanostructures portrays it as a malleable building block.

With this in mind, and considering the general desirability of using short peptide derivatives for the formation of bio-inspired nanostructures, we were motivated to derive short peptides from a pilin subunit and explore their propensity to self-assemble. As a model we selected the *Geobacter sulfurreducens* (GS) pilin, PilA (uniprotkb: A0A0J9X1J5). The GS T4Ps have gained considerable attention due to their newly discovered role as extracellular nanowires, which mediate long-range electron transport as a part of the anaerobic respiratory process^32^. A growing body of evidence suggests that GS PilA is intrinsically conductive in the context of the assembled pilus, due to a combination of sequence and packing of the subunits^26^,^27^,^28^,^33^,^34^,^35^,^36^. Structurally, GS PilA is less than half the length of most homologous pilins since the β-sheet characteristic of the globular head domain is completely lacking. This renders the α1-C region and a very short C-terminal loop as the only remaining portions of this domain^37^. The structural simplicity of GS PilA and the growing interest in this protein for potential bioelectronic applications present it as an attractive model for our purpose.

Here, we designed a 20-mer peptide based on the GS PilA sequence. This peptide can be considered as a sequence-minimized representation of the intact protein as it spans residues from both α1-N and α1-C. The designed peptide self-assembled into ordered nanofibres, which were characterized using electron and atomic force microscopy, vibrational and electronic spectroscopy and X-ray fibre diffraction. Furthermore, by following a reductionist approach, we were able to identify shorter self-assembling peptides, all of which correspond to α1-N but not to α1-C. A combination of spectroscopic methods and X-ray crystallography show that the assembly in this system results from the surprising formation of supramolecular β-sheets between α1-N-derived sequences, at the expense of their native helical conformation. In contrast, a native-like helical conformation is adopted by the α1-C-derived sequence. To our knowledge, this is the first successful assembly of peptide-based T4P-like nanostructures.

## Results

### 20-mer peptide design

The designed 20-mer peptide conjoins residues 1–8 and 22–33 of GS PilA, as presented in Table 1. Residues 1–8 were derived from the conserved α1-N region and serve to mimic the interactions within the pilus core. This specific segment was selected to impart hydrophobicity as well as for the presence of Phe1 and Glu5, whose electrostatic bond is a key interaction in the core of native T4Ps generally^20^. Residues 22–33 were derived from α1-C and exemplify the polar and aromatic content of this region. The N terminus of the peptide was left uncapped to enable the Phe1-Glu5 electrostatic bond and the C terminus was amidated to mimic the uncharged amide bond in the protein. Finally, the alanine residues present in the selected α1-C segment were substituted with the helix-nucleating α-aminoisobutyric acid (Aib)^38^. This α-methylated form of alanine possesses a highly limited range of allowed ϕ, ψ torsion angles as compared with alanine, which occupy the helical regions of the Ramachandran plot^39^. Due to this, Aib has been used to nucleate and stabilize helical conformations in various peptides, including in short peptides, which are generally less prone to adopt these conformations^9^,^40^,^41^. Therefore, to facilitate folding of the designed peptide or of its α1-C-derived segment into a helical native-like conformation, the alanine residues were substituted by Aib. A sequence-minimized form of GS PilA was thus formed.

### Monitoring the 20-mer peptide self-assembly process

**Figure 1:**
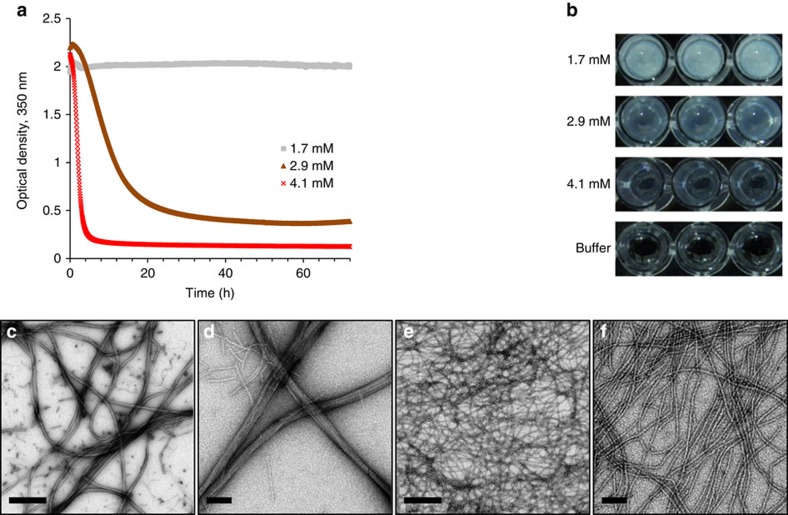
Self-assembly of the 20-mer peptide into ordered nanofibres. The nanofibres formed in phosphate buffer. (**a**) Kinetics of absorbance at 350 nm at three concentrations over a period of 72 h and (**b**) macroscopic visualization of the preparations at the end of the experiment. (**c**,**d**) TEM micrographs of the 20-mer nanofibres at 1.7 mM or (**e**,**f**) 4.1 mM. Scale bars in **c** and **e** are 500 nm; scale bars in **d** and **f** are 100 nm.

Dissolution of the peptide in phosphate buffer (10 mM, pH 7.4) resulted in an optically turbid solution. At 1.7 mM the peptide solution remained turbid over time, while at 2.9 and 4.1 mM the solution gradually cleared and at a higher rate for the higher concentration (Fig. 1a) until a semi-transparent gel-like phase was reached (Fig. 1b). Preparations of the low (1.7 mM) and high (4.1 mM) concentrations were compared at this stage by transmission electron microscopy (TEM). In both cases, flexible fibres of ∼10 nm in width and micrometers in length were observed. At the low concentration, bundling of the fibres and the formation of dense clusters were prevalent (Fig. 1c,d), while at the high concentration, networks of singular fibres were dominant (Fig. 1e,f, Supplementary Figs 1 and 2). Corresponding high-resolution scanning electron microscopy (HRSEM) imaging corroborated these findings, where at the low concentration fibre bundles were densely packed together (Supplementary Fig. 3a) and at the high concentration the surface appeared smooth and homogenous (Supplementary Fig. 3b), as can be expected from a layer of fine nanofibres. Further imaging using atomic force microcopy (AFM) indicated that the singular fibres are ∼3 nm in height (Supplementary Fig. 3c) and that the fibre bundles reach more than 20 nm in height (Supplementary Fig. 3d). The transition of a turbid peptide solution into a gel phase has been previously explained as a restructuring process where irregular aggregates with dimensions in the range of visible wavelengths transform into an array of ordered assemblies with considerably lower dimensions^42^,^43^,^44^. This explanation is applicable for the observed transition since the thin fibres seemed to appear over time at the expense of large amorphous aggregates (Supplementary Fig. 4a) and appeared to emanate from these aggregates (Supplementary Fig. 4b). The consistent turbidity values in the case of the low peptide concentration can be explained by the formation of the dense bundle clusters, which cause significant light scattering. Compared with previous reports of T4Ps morphology, the height of the 20-mer nanofibres is highly similar to that of GS T4Ps^27^,^33^, yet they are wider. Other morphological properties of the 20-mer nanofibres, including their micro-scale length, apparent flexibility and clear propensity to latterly bundle, are in line with the characteristic morphology of T4Ps.

### Secondary structure analysis of the 20-mer peptide

**Figure 2:**
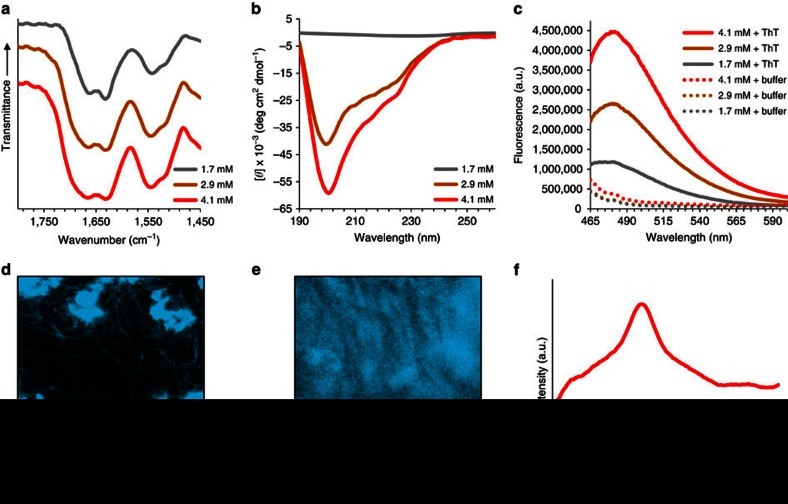
Secondary structure analysis of the 20-mer peptide in phosphate buffer. (**a**) FTIR spectra of dried peptide samples at three concentrations. (**b**) CD spectra of peptide solutions at three concentrations. (**c**) Fluorescence emission spectra of peptide preparations at three concentrations, in the presence or absence of ThT, upon excitation at 440 nm. (**d**) CLSM imaging of 1.7 mM or (**e**) 4.1 mM peptide concentrations in the presence of ThT. (**f**) Radial averaging of the X-ray fibre diffraction pattern obtained from dried stalks of the peptide at 4.1 mM. Scale bars in **d** and **e** are 10 μm.

To characterize the formed nanofibres, secondary structure analysis was performed at the above investigated concentrations. Fourier transform infrared (FTIR) spectroscopy was employed and the recorded amide I band was manifested as a doublet at 1,631 and 1,660–1,665 cm^−1^ for each of the concentrations (Fig. 2a). While the former peak position clearly indicates the presence of the β-sheet conformation^45^, the latter is ambiguous and have been assigned to β-turns, α and 3_10_ helices and random structures^45^,^46^,^47^. Deconvolution of the amide I band (Supplementary Fig. 5a; Supplementary Table 1) confirmed the substantial presence of β-sheet structures. Furthermore, it suggested that an ensemble of conformations exists in the peptide preparations, which may correspond to sub-populations or to different regions of the 20-mer. Comparable results were obtained using circular dichroism (CD) spectroscopy. While samples at 1.7 mM gave very weak CD signal in accordance with the presence of light scattering bundle clusters, peptide preparations at higher concentrations exhibited a broad negative shoulder centered at about 225 nm and a strong negative minimum near 200 nm (Fig. 2b). In good agreement with the FTIR data, secondary structures proportion estimation using multilinear regression analysis of the CD spectrum (Supplementary Fig. 5b, see Methods section) confirmed the significant presence of β-sheet structures and also highlighted the dominance of random or polyproline helix-like structures. Time-dependent CD spectra recorded at 4.1 mM (Supplementary Fig. 6) was used to monitor the gradual increase in signal during the assembly process, which occurred concomitantly with the optical clearing and fibre formation during this time period, as shown in Fig. 1a and Supplementary Fig. 4, respectively. Characteristic fluorescence emission near 480 nm upon interaction with thioflavin T (ThT), a β-sheet-specific dye^48^, provided additional evidence for the presence of β-sheets (Fig. 2c). Furthermore, ThT binding to the bundle clusters and to individual bundles as imaged using confocal laser scanning microscopy (CLSM, Fig. 2d,e) reaffirmed that this conformation exists for the peptide in its assembled state. Finally, the X-ray diffraction pattern of dried fibre stalks exhibited a ring corresponding to *D* spacing of 4.7 Å (Fig. 2f; Supplementary Fig. 7). The common interpretation of this feature, which is also consistent with the previous assays, is the distance between hydrogen-bonded β-strands^49^,^50^,^51^. The above analysis therefore suggests that the 20-mer self-assembly into nanofibres is driven by the formation of a supramolecular β-sheet. However, considering the fact that a β-breaking proline residue^52^,^53^ is present at position 9, and due to the conformational constraints imposed by the helix-inducing and β-breaking Aib residues^38^,^54^ at positions 13 and 18, it becomes clear that the entirety of the peptide cannot fold into a β-strand. It can therefore be concluded that both a β-strand-forming segment and a segment that adopts a different conformation co-exist in the peptide at the assembled state.

### 20-mer reductionist study using short peptide derivatives

To better understand the organization of the 20-mer peptide in the formed nanofibres and to potentially identify a shorter sequence that promotes supramolecular recognition as a part of the assembly process, a reductionist approach was applied. We began by deriving two shorter peptides, corresponding to the two sequences flanking Pro 9. The rationale for this segmentation was not only due to the expected β-breaking role of the proline residue, but also because the sequence N-terminally to this residue originates from α1-N in GS PilA, while the sequence C-terminally to it originates from α1-C. Since Pro 9 terminates α1-N in GS PilA and directly precedes α1-C, it was included in both of the derived peptides, resulting in an N-terminal 9-mer and a C-terminal 12-mer (Table 1). Indeed, these two peptides differed in their propensity to self-assemble and exhibited distinct folding.

**Figure 3:**
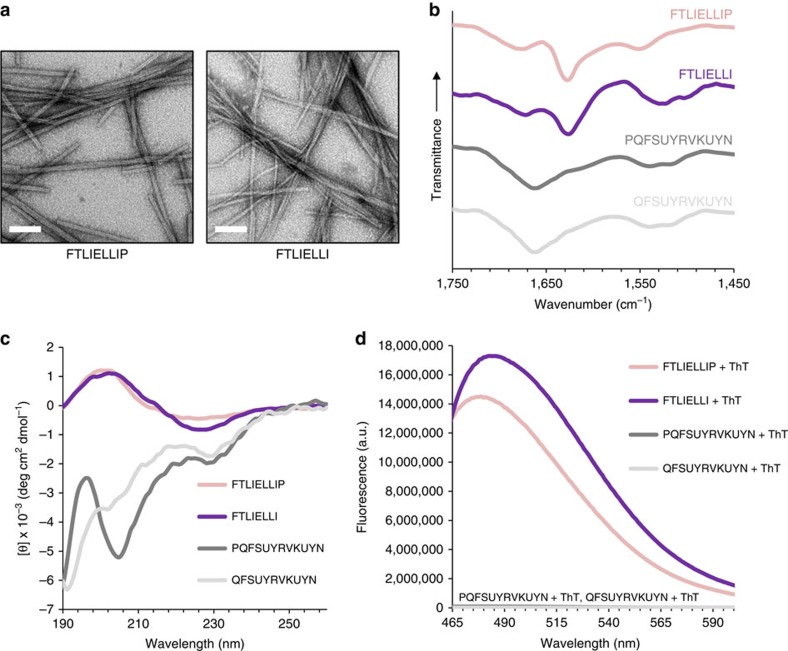
Structural analysis of the self-assembling 9-mer & 8-mer and the non-assembling 12-mer & 11-mer peptides. The self-assembling peptide sequences are FTLIELLIP and FTLIELLI, and the non-assembling peptide sequences are PQFSUYRVKUYN and QFSUYRVKUYN. (**a**) TEM micrographs of the 9-mer and 8-mer nanofibres. (**b**) FTIR spectra of dried peptide samples. (**c**) CD spectra of the peptide solutions. (**d**) Fluorescence emission spectra of the peptides, in the presence of ThT, upon excitation at 440 nm. For all assays, peptides were prepared at 2.9 mM in phosphate buffer, except for CD spectroscopy of the 12-mer and 11-mer, where the peptides were diluted with buffer to 0.725 mM. Scale bars in **a** are 100 nm.

**Figure 4:**
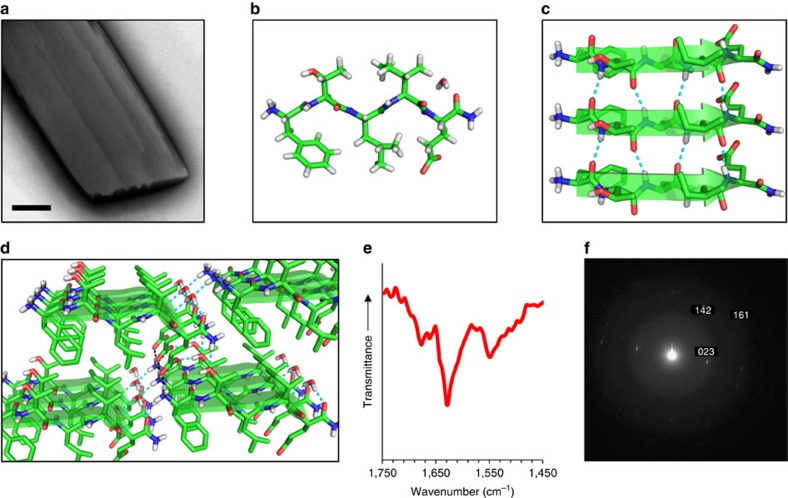
Structure of the 5-mer (FTLIE) microcrystals. (**a**) TEM micrograph of an individual microcrystal grown in phosphate buffer at a concentration of 2.9 mM. (**b**) View of the asymmetric unit as determined for 5-mer single-crystals by XRD, showing a single peptide molecule and a water molecule. (**c**) Crystal packing of three peptide molecules along the crystallographic *a* axis. The peptide molecules are organized as a supramolecular parallel β-sheet. (**d**) Extended crystal packing, showing a network of hydrogen bonds and electrostatic interactions between β-sheets along the crystallographic *b* and *c* axes. For panels **c**,**d**, hydrogen bonds and electrostatic interactions are shown as dashed cyan and black lines, respectively. (**e**) FTIR spectrum of microcrystals grown in phosphate buffer at a concentration of 11.6 mM. (**f**) SAED pattern of an individual microcrystal grown in buffer at a concentration of 16 mM. Reflections are indexed in relation to the single crystal structure as obtained by XRD. Scale bar in **a** is 500 nm.

As with the 20-mer peptide, the investigation of the two derived peptides was performed in phosphate buffer. The 9-mer peptide self-assembled into linear nanofibres of ∼3 nm in width and microns in length which appeared to bundle, thus reaching few tens of nm in width as evident by TEM (Fig. 3a) and more than 10 nm in height as evident by AFM (Supplementary Fig. 8a). This occurred at all three concentrations as tested for the 20-mer. The 9-mer FTIR spectrum presented a sharp amide I band at 1,628 cm^−1^ (Fig. 3b), consistent with a predominant presence of β-sheets^45^. This was supported by a CD spectrum with a negative minimum near 225 nm and a positive maximum near 200 nm (Fig. 3c). This 225 nm minimum is red-shifted compared to the characteristic β-sheet minimum near 217 nm (ref. 55), yet similar spectra were previously reported in other supramolecular β-sheet peptide assemblies^56^,^57^. Lastly, ThT binding further supported the formation of β-sheets by the 9-mer (Fig. 3d). In contrast, the 12-mer peptide did not self-assemble under the experimental conditions as nanofibres or other assemblies were not detected by TEM imaging. The CD spectrum of the 12-mer peptide displayed negative minima at 228 and 205 nm (Fig. 3c). A highly similar CD spectrum was previously reported for a sequential Aib-Alanine peptide which, based on the minima positions and the ratio of their intensities, was determined to be a partially folded α-helix^58^. The FTIR spectrum of the 12-mer contained an amide I band at 1,662 cm^−1^ (Fig. 3b); in light of the CD data and considering the typical assignment of such band, this signal can be attributed to the presence of a helical conformation^45^. It is also worth noting that ThT binding was not detected for this peptide (Fig. 3d). Similar results were obtained for an 11-mer lacking the N-terminal proline (Fig. 3), yet the propensity of this peptide to adopt a helical conformation was lower, as evident mostly from the diminished 205 nm band in the CD spectrum. The lower propensity is in line with the highly unfavorable positioning of glutamine instead of proline as the first residues in a helix or at helix-preceding positions^59^. The results discussed thus far clearly reveal that the α1-N-derived sequence self-assembles into β-sheet-based nanofibres, while the sequences derived from α1-C, especially the 12-mer, fold into a helical structure which is not capable of self-assembly. To identify a more specific sequence which promotes supramolecular recognition in this system, several shorter peptides were derived from the α1-N 9-mer and investigated in phosphate buffer. First, Pro 9 was removed, resulting in an 8-mer (Table 1). This peptide also self-assembled into nanofibres extending to a length of microns as observed by TEM and AFM imaging (Fig. 3a; Supplementary Fig. 8b), yet these appeared more flexible, less prone to form bundles, and also wider and flatter compared with the 9-mer assemblies, presenting an approximated width and height of about 10 and 3 nm, respectively. Secondary structure analysis of these assemblies by FTIR, CD and ThT assays gave very similar results to those obtained for the 9-mer (Fig. 3b–d), presenting high structural similarity between the two peptides. Next, the three hydrophobic amino acids at the C terminus were removed to obtain a 5-mer (Table 1), which spontaneously formed thin plate-shaped microcrystals in buffer (Fig. 4a). Single crystals suitable for diffraction were grown in 10–25% 2,2,2-trifluoroethanol (TFE) and the crystal structure was determined by X-ray diffraction (XRD) at 0.72 Å resolution. The determined structure belongs to a triclinic crystal system, space group *P*1, with one peptide molecule and one water molecule per asymmetric unit (Fig. 4b; Supplementary Fig. 9; Supplementary Data 1). The crystal packing clearly shows that the 5-mer tends to organize in supramolecular parallel β-sheets, predominantly stabilized by the π–π stacking interactions between phenylalanine side chains (Fig. 4c,d), which propagate along the morphological long axis of the crystal (Supplementary Fig. 10). These β-sheets are further packed together along the perpendicular axes by electrostatic interactions between the Phe1 N-termini and the Glu5 side chains, as well as by a hydrogen bonding network that includes the N- and C-termini, the Thr2 and Glu5 side chains and water molecules (Fig. 4d; Supplementary Table 2). Confirming that this structure exists in microcrystals grown in buffer, FTIR spectra of such preparations exhibited a sharp amide I band at 1,630 cm^−1^ (Fig. 4e) and a selected area electron diffraction (SAED) pattern of individual microcrystals fitted the lattice parameters as obtained by XRD (Fig. 4f; Supplementary Table 3). The structure of the 5-mer therefore shows that the propensity of the investigated α1-N sequences to form supramolecular β-sheets, and hence their capability to function in supramolecular recognition, can be mapped to this short N-terminal sequence.

The propensity to form supramolecular β-sheets is maintained even in the case of a 4-mer lacking the C-terminal glutamate (Table 1). This peptide self-assembled in phosphate buffer to form bundling nanofibres and nanoribbons with dimensions of up to tens of nanometers in width, approximately 1.5 nm in height and microns in length as was evident by TEM and AFM imaging (Supplementary Fig. 11a,b). The FTIR spectrum of this peptide revealed a bifurcate amide I band with peaks at 1,620 and 1,666 cm^−1^ (Supplementary Fig. 11c), indicating the presence of β-sheet and β-turn or random conformation, respectively^45^,^46^,^47^. Consistent with the FTIR data, the CD spectrum of this peptide gave a strong negative minimum at 230 nm (Supplementary Fig. 11d). Similar spectra have been attributed to β-sheet or β-turn structures in very short peptides that self-assemble into supramolecular nanofibres^60^,^61^,^62^. Finally, ThT binding was well consistent with the presence of β-sheets (Supplementary Fig. 11e). This data therefore shows that the 4-mer assemblies are supramolecular β-type nanofibres, and are thus similar in their general organization to the 5-mer microcrystals. However, in the absence of Glu5, which participates in hydrogen and electrostatic bonds that stabilize the crystal growth in the axes perpendicular to the β-sheet axis, the 4-mer assemblies manifest as thinner assemblies as compared with the 5-mer microcrystals. To further show the strong propensity to self-assemble in peptides derived from the α1-N domain in contrast to peptides derived from the α1-C region, two short α1-C control peptides were derived and investigated in phosphate buffer. The peptides are the two most hydrophobic 5-mers as per the Fauchere/Pliska scale^63^ that can be derived from the remaining portion of α1-C, C-terminally to the investigated 12-mer and 11-mer (Supplementary Fig. 12a). For consistency with the alanine-Aib substitution in 12-mer and 11-mer peptides, Aib-containing analogues of the control 5-mers were investigated as well. In all cases, self-assembly was not detected using electron microscopy. Furthermore, secondary structure analysis showed that the control peptides have a random structure as was evident by the 1,648 or 1,660 cm^−1^ amide I band position in the FTIR spectrum (Supplementary Fig. 12b) and a single minimum at 196–200 nm in the CD spectrum (Supplementary Fig. 12c). Lastly, ThT binding was not detected for the control peptides, confirming the absence of β-sheet structures (Supplementary Fig. 12d). It is therefore concluded that short sequences derived from the evolutionary conserved α1-N polymerization domain of a pilin tend to adopt the β-sheet conformation, at the expense of their native helical conformation, and self-assemble into high aspect ratio structures at the micro- or nano-scale. This phenomenon is not observed for peptides derived from the α1-C region.

## Discussion

In this work, we have shown that T4P-like nanostructures can be obtained by using peptide self-assembly as a strategy for their formation. With this general strategy, the inability to assemble pilin monomers into T4P-like nanofibres *in vitro* can be circumvented. Furthermore, as in the design of other bio-inspired nanostructures, the established synthesis procedures and commercial availability of peptides highlight them as the building block of choice for the formation of T4P-like nanostructures. The investigated 20-mer peptide is a minimized form of the GS pilin subunit, encompassing two distinct sequences from the evolutionary conserved polymerization domain and the functionality-related variable region of the protein. The reductionist approach employed in this study showed that the N-terminal segment of the 20-mer, which corresponds to the GS pilin N-terminal polymerization domain α1-N, adopts a β-type conformation. While this segment, as a part of the conserved α1-N domain, natively adopts an α-helical conformation in the GS pilin and in pilin proteins generally, homologous sequences can form α-helices or β-strands in a variety of other proteins (Supplementary Table 4). This suggests that the investigated α1-N sequences can be conformationally permissive and that the adoption of a particular conformation is a context-dependent event. Specifically, the membrane environment pertinent to pilin translation and polymerization *in vivo* is a factor likely to promote the helical conformation of α1-N, yet outside of this environment, a β-type conformation may arise. In the investigated system, the β-strand conformation is indeed adopted by the α1-N derived sequences. The ensuing supramolecular β-sheet configuration, which forms in the process of self-assembly, is a property shared by the different investigated assemblies. The totality of the data, and particularly the high-resolution structure of the α1-N 5-mer, indicates that the β-sheet interaction propagates along the morphological long axis of the assemblies, leading in all cases to their elongated shape. However, the width and height of the different assemblies varies, with considerable differences between the 4-mer nanofibres and nanoribbons, the 5-mer microcrystals, and the nanofibres formed by the longer peptides. Both the 4-mer and 5-mer peptides form wider assemblies that can form due to stable interactions between individual β-sheets in at least one plane perpendicular to their propagation direction. This is considerably more pronounced in the case of the 5-mer since Glu5 upholds multiple stabilizing interactions in both axes perpendicular to the β-sheet direction, as evident from the crystallographic data. In contrast, the longer peptides form fibres with low nanometric width and height. This difference can be regarded as the result less stable interfaces in the axes perpendicular to the axis of β-sheet propagation. Additionally, non-specific interactions between hydrophobic side-chains, which may protrude from the fibres as a part of the β-sheet arrangement, could lead to fibre bundling and clustering and thus prevent additional ordered growth perpendicularly to the β-sheet propagation axis.

The supramolecular β-sheet configuration can be considered as the result of alternative molecular recognition between α1-N sequences, as compared with the configuration of the respective domain in native T4P core. While the existing structural model for T4P suggests an architecture based on spiraling helix bundles^20^, our data raises the possibility that α1-N sequences may in fact form supramolecular β-sheets in the core of T4Ps *in vivo*. This may be plausible since the existing approach for the structural elucidation of T4Ps utilizes the fitting of a high-resolution structure of the pilin monomer into a lower-resolution cryo-electron microscopy envelope of the intact nanofibre^64^; while this approach is powerful, it is limited in providing atomic resolution data on the interface between monomers^65^, and does not enable the atomistic study of intact T4Ps. Furthermore, taking into account that the mechanism of T4Ps polymerization is still not fully understood, it may therefore be possible that in the assembled state, the pilin subunit α1-N domain diverges somewhat from its monomeric structure.

**Figure 5:**
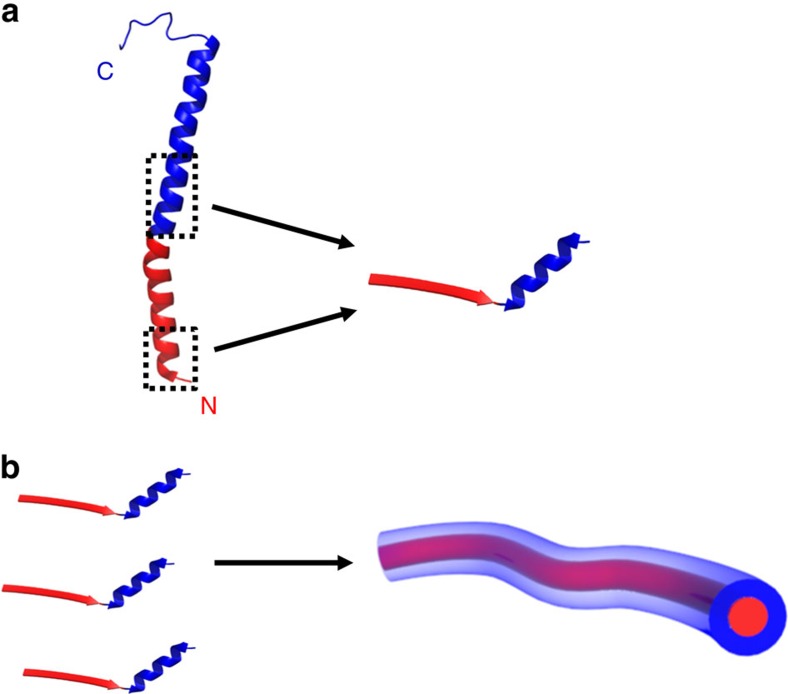
Illustration of the strategy for mimicking a T4P nanofibre using peptide self-assembly. (**a**) By joining a segment from the evolutionary conserved N-terminal polymerization domain α1-N (red) with a segment from the functionality-related C-terminal region α1-C (blue), a sequence-minimized representation of the pilin protein is formed. In this peptide, the former segment adopts the β-strand conformation while the latter segment maintains a native-like helical conformation. (**b**) This peptide self-assembles into nanofibres via the formation of a supramolecular β-sheet between α1-N-derived sequences, which can be considered as an alternative configuration to the one adopted by the respective domain in the native T4P core. Structure of the intact pilin as given in panel **a** was taken from PDB 2M7G (ref. 37).

In contrast to the N-terminal α1-N-derived segment of the 20-mer, the C-terminal α1-C-derived segment does not self-assemble and folds into a helical conformation, resembling its native conformation. In line with the role of α1-N in the polymerization of native T4P, and likely due to the higher hydrophobicity of α1-N as compared with α1-C, peptides derived from the former present a clear propensity to self-assemble, as opposed to peptides derived from the latter. Therefore, to obtain a short pilin-derived building block that can both self-assemble and display a native-like conformation, α1-N and α1-C derived segments can be conjoined in a single peptide (Fig. 5). Further study of the obtained nanofibres may therefore reveal functionalities similar to those of the native GS T4P and can potentially lead to the development of new peptide-based bioelectronic materials. Our strategy may also be used in the design of other T4P-derived building blocks for mimetic nanostructured biomaterials, where specific segments with functional importance in other pilins are conjoined with an assembly-driving pilin-derived sequence.

## Methods

### Preparation of peptide assemblies

All peptides were synthesized by Pepmic Co., Ltd. (Suzhou, China), except for the N-terminal 4-mer peptide, the C-terminal 11-mer and the four C-terminal control peptides which were synthesized by Peptron Inc. (Daejeon, South Korea). The peptides were purified to at least 95%, and their identity was confirmed by mass spectrometry. For the formation of the 20-mer assemblies, lyophilized peptide was dissolved to the desired concentration in 10 mM potassium phosphate buffer (pH 7.4) under vigorous vortexing (∼30 s). For the other peptides, unless otherwise stated, dimethyl sulfoxide (DMSO) was used to prepare a concentrated stock solution (50 mg ml^−1^) which was then diluted with buffer to a concentration of 2.9 mM and a final DMSO percentage of ∼4–9%. For all assays, unless otherwise stated, all peptide preparations were incubated for 3–4 days before examination.

### Turbidity analysis

Turbidity analysis for the 20-mer peptide preparations was conducted by preparing fresh solutions at concentrations of 1.7, 2.9 or 4.1 mM in buffer. Then, 200 μl aliquots were pipetted into a 96-well plate, sealed using a Breathe-Easy sealing membrane (Sigma Aldrich, Rehovot, Israel), and absorbance at 350 nm was measured over time, starting less than 10 min after preparation of the peptide solutions. All measurements were performed using a Synergy HT plate reader (Biotek, Winooski, VT, USA) at 25 °C.

### Transmission electron microscopy

TEM imaging was performed by applying 10 μl samples onto 400-mesh copper grids covered by a carbon-stabilized Formvar film (SPI, West Chester, PA, USA). The samples were allowed to adsorb for 2 min before excess fluid was blotted off. Negative staining was then achieved by depositing 10 μl of 2% uranyl acetate on the grid for 2 min before blotting off excess fluid. Micrographs were recorded using a Tecnai 12 electron microscope (FEI, Tokyo, Japan) operating at 120 kV.

### High-resolution scanning electron microscopy

HRSEM imaging was performed by applying 5 μl solution samples on glass coverslips, allowing them to dry under ambient conditions overnight and coating the samples with Cr. Micrographs were recorded using a JSM-6700F FE-SEM (JEOL, Tokyo, Japan) operating at 2 kV.

### Atomic force microscopy

AFM imaging was performed by depositing 10 μl solutions onto freshly cleaved V1 grade mica (Ted Pella, Redding, CA, USA). The samples were allowed to dry under ambient conditions. Images were obtained with AIST-NT Smart AFM system in non-contact (tapping) mode using 100 μm long silicon nitride cantilevers (OMCL-RC800PSA-W, Olympus, Japan) with resonance frequency of 70 kHz. The images were analysed and visualized using the WSxM imaging software^66^ (Nanotec Electronica S.L, Madrid, Spain).

### Fourier transform infrared spectroscopy

FTIR spectroscopy was performed with 30 μl samples of peptide solutions, deposited onto disposable KBr infrared sample cards (Sigma-Aldrich, Rehovot, Israel), which were then allowed to dry under vacuum. Transmission infrared spectra were collected using a nitrogen-purged Nexus 470 FTIR spectrometer (Nicolet, Offenbach, Germany) equipped with a deuterated triglycine sulfate (DTGS) detector. Measurements were made by averaging 64 scans in 4 cm^−1^ resolution. The amide I region was deconvoluted by the second derivative method using the Peakfit software version 4.12 (Systat Software Inc., San Jose, CA, USA).

### CD spectroscopy

CD spectroscopy was performed for the self-assembling peptides at the concentration of self-assembly without further dilution. The non-assembling 12-mer and 11-mer peptides were diluted with buffer to a concentration of 0.725 mM, and the non-assembling C-terminal control peptides were similarity diluted to a concentration of 1.45 mM. For the peptides prepared using DMSO (see Preparation of Peptide Assemblies above), 1,1,1,3,3,3-hexafluoro-2-propanol (HFIP) was used instead of DMSO. CD spectra were collected with a Chirascan spectrometer (Applied Photophysics, Leatherhead, UK) fitted with a Peltier temperature controller set to 25 °C, using quartz cuvettes with an optical path length of 0.1 or 0.01 mm (Hellma Analytics, Müllheim, Germany). Absorbance was kept at the linear range of the instrument during all measurements. Data acquisition was performed in steps of 1 nm at a wavelength range of 190 to 260 nm with a spectral bandwidth of 1.0 nm and an averaging time of 3 s. The spectrum of each sample was collected three times and averaged. Spectra were corrected in baseline with the spectra of buffer and HFIP at the corresponding percentage, which were similarly collected. Data processing was done using Pro-Data Viewer software (Applied Photophysics, Leatherhead, UK); processing and normalization to mean residue ellipticity (MRE) was performed using standard calculation^67^. Non-constrained multilinear regression analysis of the 20-mer data was performed by using the Brahms and Brahms data set^68^, with the reference for the random coil structure substituted by the collagen reference as reported by Wallace and co-workers^69^. Analysis was performed using the SigmaPlot software (Systat Software Inc.).

### Thioflavin T fluorescence

For ThT fluorescence spectra acquisition, peptide solutions were diluted at a 1:1 ratio (v/v) with a solution of 100 μM ThT in buffer including the appropriate percentage of DMSO (0–9%), transferred immediately into an ultra-micro fluorescence cuvette with an optical path length of 10 mm (Hellma Analytics, Müllheim, Germany) and measured using a Fluorolog-3 spectrofluorometer (Horiba Jobin Yvon, Edison, NJ, USA). The excitation wavelength was set to 440 nm and emission was recorded between 465 and 600 nm, with excitation and emission slits of 5 nm. Spectra of peptides in ThT were corrected in baseline with ThT solutions in buffer that included the appropriate percentage of DMSO. For CLSM imaging, samples were prepared similarly and incubated 3 h unexposed to light. Imaging of wet samples was done using a LSM 510 Meta confocal laser scanning microscope (Carl Zeiss, Oberkochen, Germany) at excitation and emission wavelengths of 458 and 485 nm, respectively.

### X-ray fibre diffraction

Dried stalks of the 20-mer nanofibres were prepared as described before^70^, by suspending ∼15 μl of 4.1 mM of peptide solutions in buffer between the ends of wax-capped glass capillaries with an outer diameter of 1.5 mm. The stalks were dried over a period of 24 h in the presence of buffer solution inside a sealed container, and then further dried overnight after removal of the buffer. Measurements were conducted using a Pilatus 300 K detector (Dectris, Baden, Switzerland) and a GeniX Low Divergence (Xenocs, Sassenage, France) Cu Kα radiation source set-up with scatterless slits^71^. Two-dimensional frames were radially averaged with and radially integrated using Matlab (MathWorks, USA) based procedures (SAXSi). Structural analysis was performed using X+ software^72^.

### Single crystal X-ray structure determination

5-mer peptide crystals suitable for XRD were grown in batch by dissolving lyophilized powder of the peptide in 10–25% TFE to a concentration of 161 uM under vortexing. A colourless needle of dimensions 0.15 × 0.08 × 0.05 mm was transferred to Hampton Paratone oil and mounted on a Hampton loop and flash frozen in liquid nitrogen. XRD data were collected at 100 K at the European Synchrotron Radiation Facility station ID29 (ref. 73; ESRF ID29) with radiation *λ*=0.70 Å using MXCube^74^. Data were collected as 0.5° frames, 0.4 s deg^−1^. A total of 720 frames were collected. Frames were processed with EDNA software package^75^. Data were collected to a 2*θ*_max_=49.66° with limiting indices −6≤h≤ 6, −16≤k≤16, −20≤l≤20 a total of 9,272 reflections, of which 3,773 were independent, *R*_int_ 0.044. The structure was solved and refined using SHELX-2013 in triclinic space group *P*1 with Z=1. Atoms were refined anisotropically with the exception of hydrogen atoms that were placed in calculated positions and refined in riding mode. Full-matrix least-squares refinement based on *F*^2^ with SHELXL-2013 on 448 parameters with 18 restraints gave final *R*_1_=0.0470 (based on *F*^2^) for data with I>2σ(I), and w*R*_2_=0.1257 on 3,773 reflections, goodness-of-fit on *F*^2^=1.029, largest electron density peak 0.71 e Å^−3^, and the largest hole –0.78 e Å^−3^. Crystal data collection and refinement parameters are given in Supplementary Table 3. Coordinates for the structure can be found in the supplemental crystallographic information file (Supplementary Data 1). Unit cell measurement in respect to crystal morphology was performed using a Bruker KappaApexII system equipped with a sealed-tube MoK(alpha) radiation source. The crystal was coated in Hampton Paratone oil, mounted on a MiTeGen loop and flash frozen in liquid nitrogen. Data were measured at 100 K as omega scans. The unit cell was determined and the faces indexed with Bruker Apex2 Suite.

### Electron diffraction

Solution of the 5-mer peptide was prepared by dissolution of the powder with HFIP to a concentration of 322.2 mM and dilution with buffer to a concentration of 16.1 mM and 5% HFIP. Solution samples were deposited on carbon-coated TEM grids, blotted and dried under ambient conditions. Electron diffraction experiments were performed using a Tecnai 12 electron microscope (FEI, Tokyo, Japan), where the samples were cooled to liquid nitrogen temperature using a Gatan 626 cryoholder (Gatan GmbH, Munich, Germany). SAED pattern was obtained using low electron dose imaging at acceleration voltage of 120 kV and recorded with a Gatan MultiScan 791 CCD camera. Analysis was performed with the Gatan DigitalMicrograph 3.1 software package.

### Data availability

The data that support the findings of this study are available from the corresponding author upon reasonable request. The X-ray crystallographic coordinates for the atomic structure reported in this study have been deposited at the Cambridge Crystallographic Data Centre (CCDC) under deposition number CCDC 1487696. This data can be obtained free of charge from the Cambridge Crystallographic Data Centre via www.ccdc.cam.ac.uk/data_request/cif.

## Additional information

**How to cite this article**: Guterman, T. *et al.* Formation of bacterial pilus-like nanofibres by designed minimalistic self-assembling peptides. *Nat. Commun.* **7**, 13482 doi: 10.1038/ncomms13482 (2016).

**Publisher's note:** Springer Nature remains neutral with regard to jurisdictional claims in published maps and institutional affiliations.

## Supplementary Material

Supplementary Information Supplementary Figures 1-12, Supplementary Tables 1-4 and Supplementary References

Supplementary Data 1 Crystallographic Information File

## Figures and Tables

**Table 1:** The main GS PilA peptides investigated*.

**Peptide**	**Sequence**	**Position in GS PilA**	**Assemblies**
20-mer	FTLIELLIPQFSUYRVKUYN†	1-8,22 (α1-N), 23-33 (α1-C)	Nanofibres
12-mer	PQFSUYRVKUYN†‡	22 (α1-N), 23-33 (α1-C)	None
11-mer	QFSUYRVKUYN†‡	23-33 (α1-C)	None
9-mer	FTLIELLIP	1-8,22 (α1-N)	Nanofibres
8-mer	FTLIELLI	1-8 (α1-N)	Nanofibres
5-mer	FTLIE	1-5 (α1-N)	Microcrystals
4-mer	FTLI	1-4 (α1-N)	Nanofibres, Nanoribbons

^*^All peptides are C-terminally amidated in accordance with their positions in GS PilA.

^†^U denotes α-aminoisobutyric acid (Aib).

^‡^The peptide is N-terminally acetylated in accordance with its position in GS PilA.
