# Supplementary material for: Formation of bacterial pilus-like nanofibres by designed minimalistic self-assembling peptides
Source: Nat Commun. 2016 Nov 17;7:13482. doi: 10.1038/ncomms13482 (PMC5473601; doi:10.1038/ncomms13482)
Supplement: Supplementary Information — Supplementary Figures 1-12, Supplementary Tables 1-4 and Supplementary References [file ncomms13482-s1.pdf]

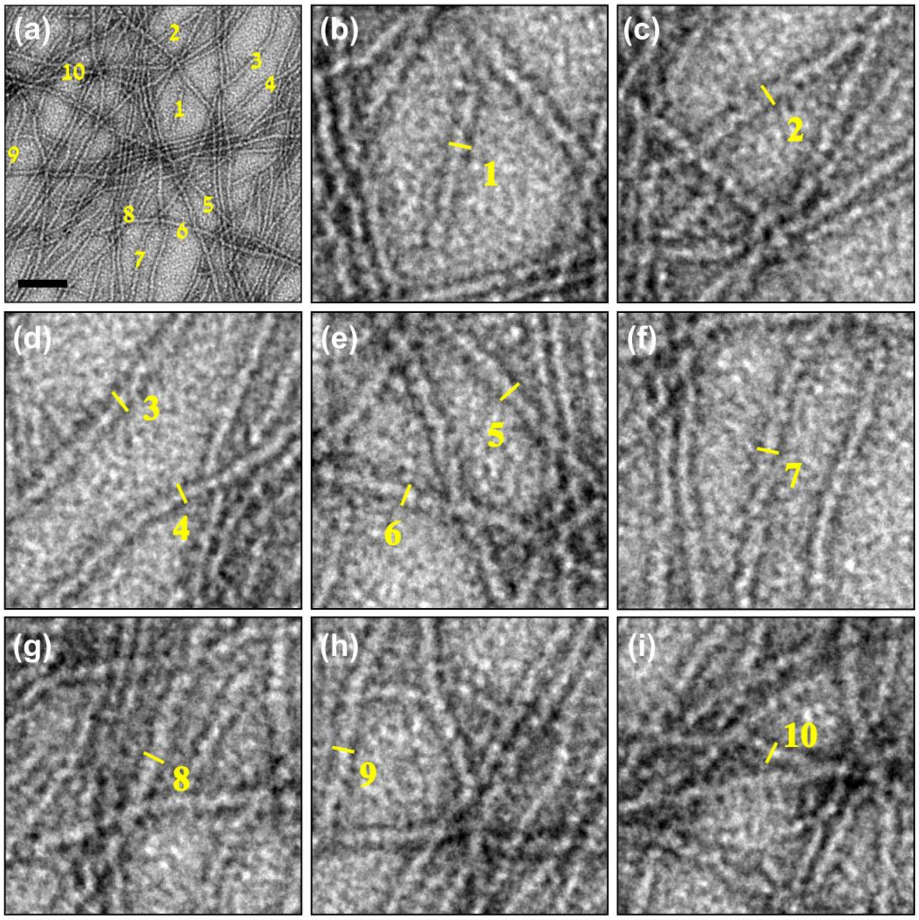

Supplementary Figure 1. Width measurement of the 20-mer nanofibers. (**a**) TEM micrograph of the 20-mer nanofibers at 4.1 mM, prepared in phosphate buffer. The 10 individual nanofibers measured are numbered in yellow. Scale bar is 100 nm. (**b-i**) Magnified view (400%) of the measured nanofibers. For each nanofiber, width in nm was measured along the yellow line. The measured widths in respect to the numbering of the nanofibers are as follows: 9.7, 10, 10.9, 9, 11, 10, 9.7, 9.9, 10, 9.8 nm. The averaged width of the nanofibers is 10±0.6 nm.

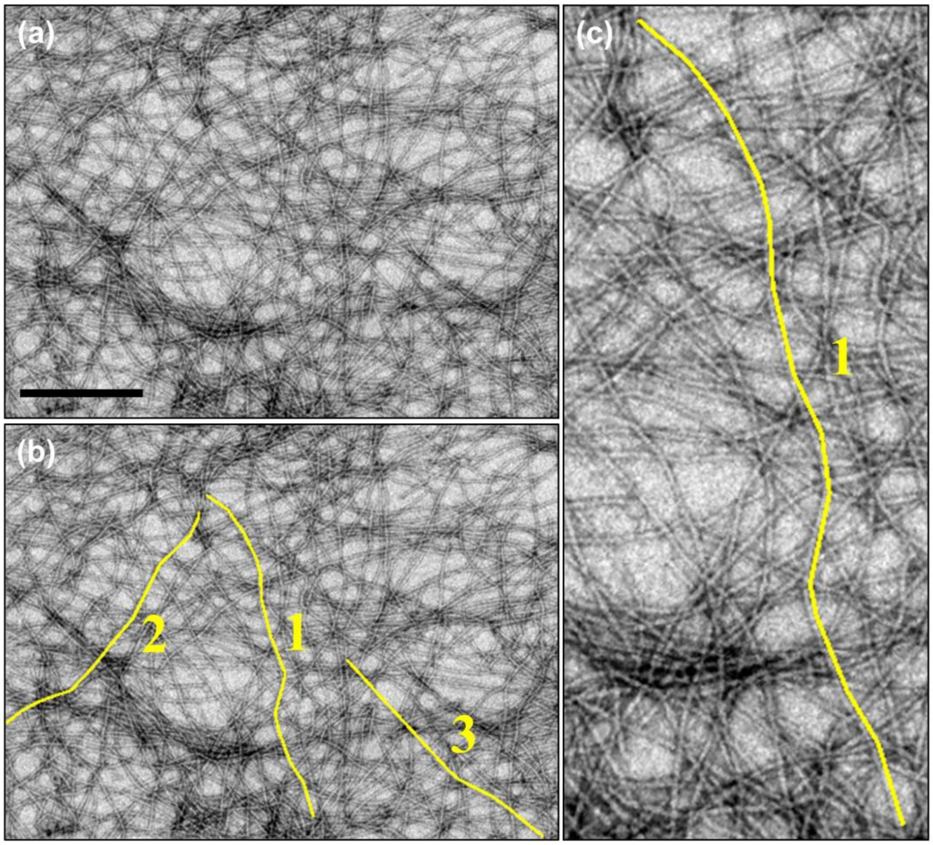

Supplementary Figure 2. Length measurement of the 20-mer nanofibers. (**a**) TEM micrograph of the 20-mer nanofibers at 4.1 mM, prepared in phosphate buffer. Scale bar is 500 nm. (**b**) Length measurements, superimposed on the micrograph given in panel **a**. The three individual nanofibers measured are numbered in yellow. For each nanofiber, length in µm was measured along the yellow line. The measured lengths in respect to the numbering of the nanofibers are as follows: 1.44, 1.2, 1.04 µm. (**c**) Magnified view (~200%) of the nanofiber numbered as 1 in panel **a**. The yellow line follows the right edge of the nanofiber.

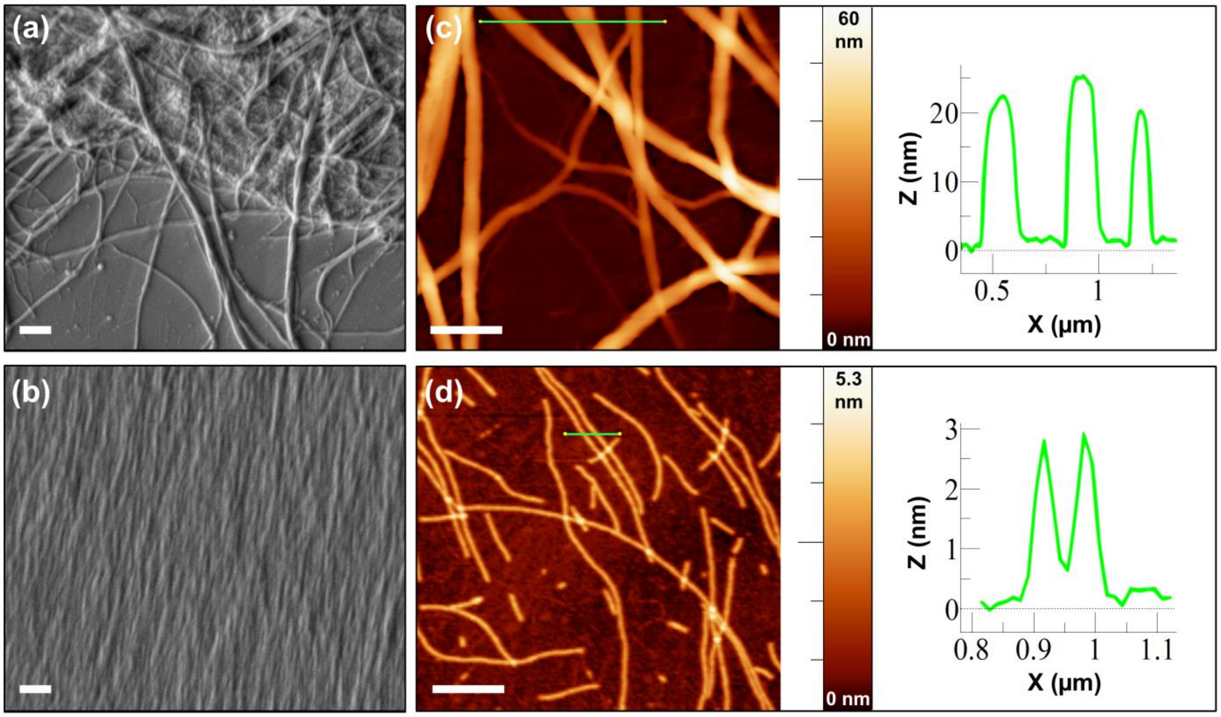

Supplementary Figure 3. **HRSEM and AFM imaging of the 20-mer peptide nanofibers.** HRSEM micrographs of the 20-mer peptide nanofibers at (**a**) 1.7 mM and at (**b**) 4.1 mM. AFM images of the 20-mer peptide nanofibers at (**c**) 1.7 mM and at (**d**) 4.1 mM. For panels **c–d**, example height profile along the green line is shown on the right. Scale bars in **a–d** are 400 nm.

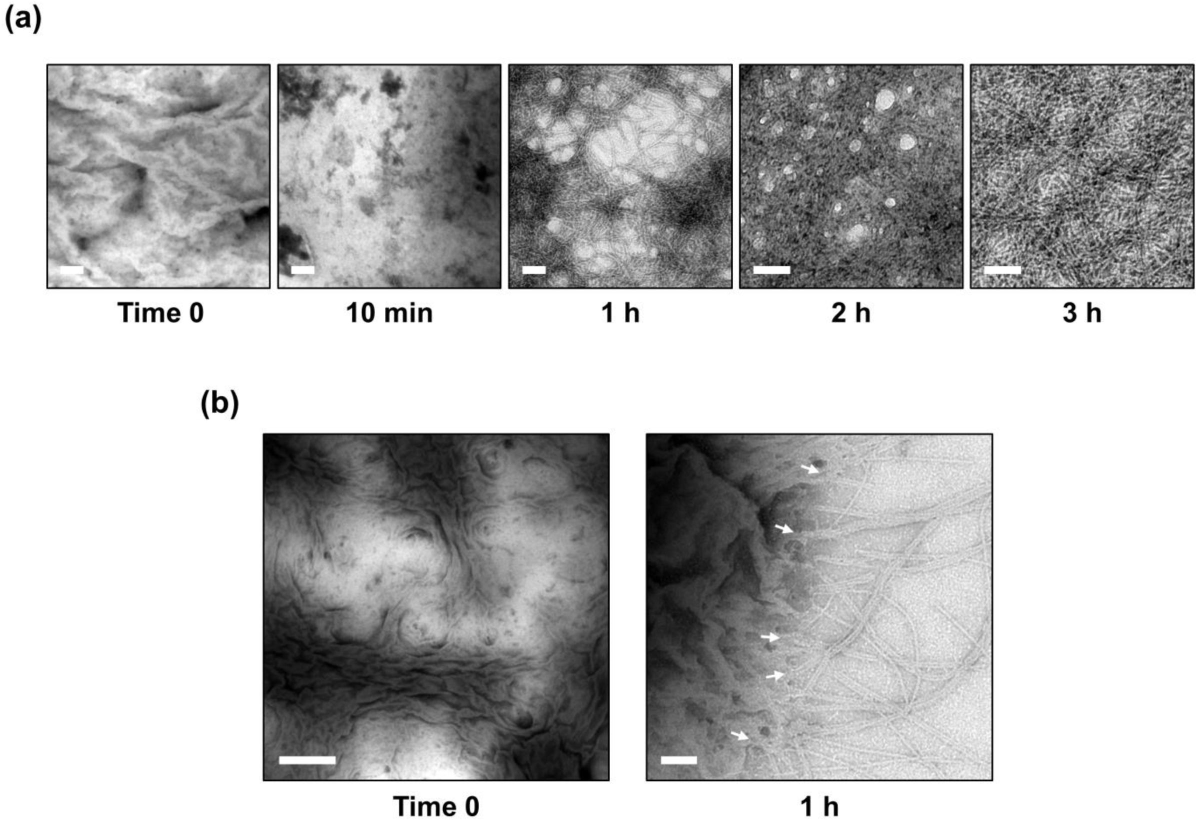

Supplementary Figure 4. Monitoring the 20-mer peptide assembly using TEM imaging. (**a**) TEM micrographs of a 20-mer preparation taken at different time points following dissolution of the peptide in phosphate buffer at a concentration of 4.1 mM. (**b**) TEM micrographs showing large irregular aggregates of the peptide at Time 0, and the apparent emanation of nanofibers from such aggregates later in the assembly process. White arrows are drawn to guide the eye. Scale bars in **a** are 100 nm; scale bars in **b** are 1 µm and 100 nm for Time 0 and 1 h, respectively.

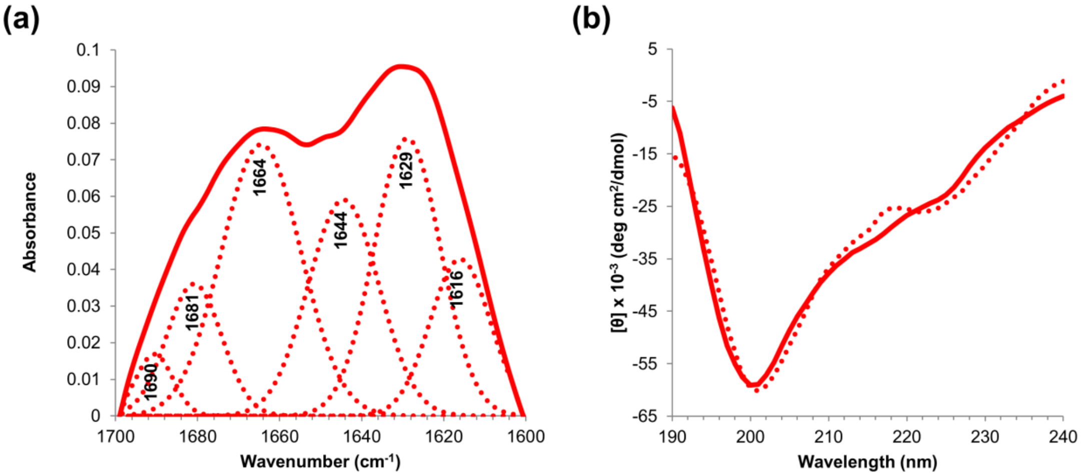

Supplementary Figure 5. Secondary structure proportion estimation of the 20-mer peptide. Deconvolution of (**a**) FTIR and (**b**) CD spectra of the 20-mer peptide, prepared at 4.1 mM in phosphate buffer. For FTIR spectrum deconvolution, the component bands result from Gaussian curve-fitting of the experimental amide I band using the second derivative method, r^2>0.997. For CD spectrum deconvolution, a non-constrained multilinear regression fitting was performed, r^2>0.97. For both panels, solid line represents experimental data and dashed line represents a fitted curve. The calculated relative percentage of secondary structures is given in Supplementary Table 1.

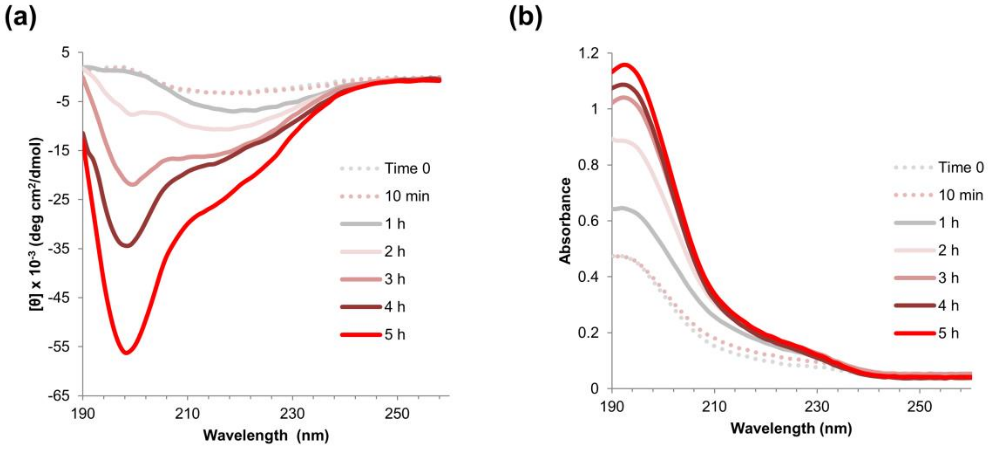

Supplementary Figure 6. Monitoring the 20-mer peptide assembly using time-dependent CD. (**a**) CD spectra of 4.1 mM peptide preparation in phosphate buffer recorded at different time points. (**b**) Respective absorbance spectra in the far-UV region.

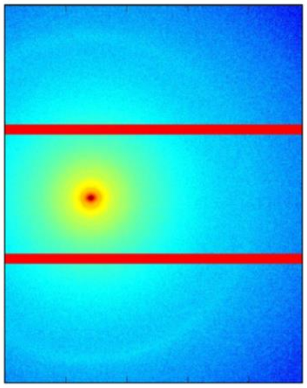

Supplementary Figure 7. X**-ray fiber diffraction pattern obtained from a dried stalk of the 20-mer peptide.** Dried stalk was prepared from peptide solution at 4.1 mM in phosphate buffer. Horizontal red lines mask the boundary between the three modules of the detector.

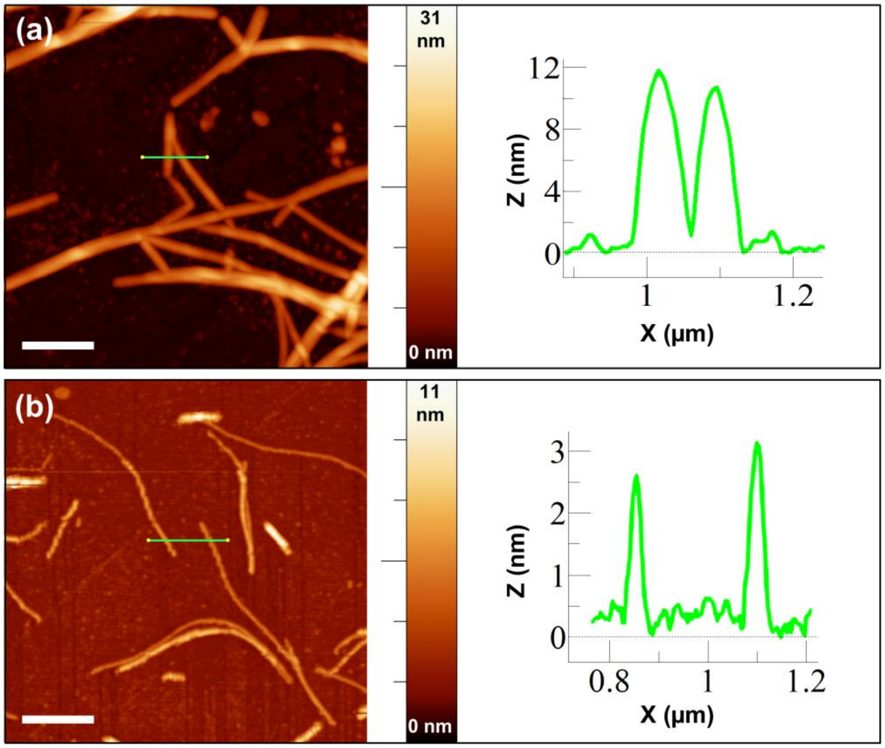

Supplementary Figure 8. AFM imaging of the 9-mer (FTLIELLIP) and 8-mer (FTLIELLI) nanofibers. (**a**) AFM image of the 9-mer and (**b**) 8-mer nanofibers. For both panels, example height profile along the green line is shown on the right. Peptides were prepared at 2.9 mM in phosphate buffer. Scale bars in **a–b** are 400 nm.

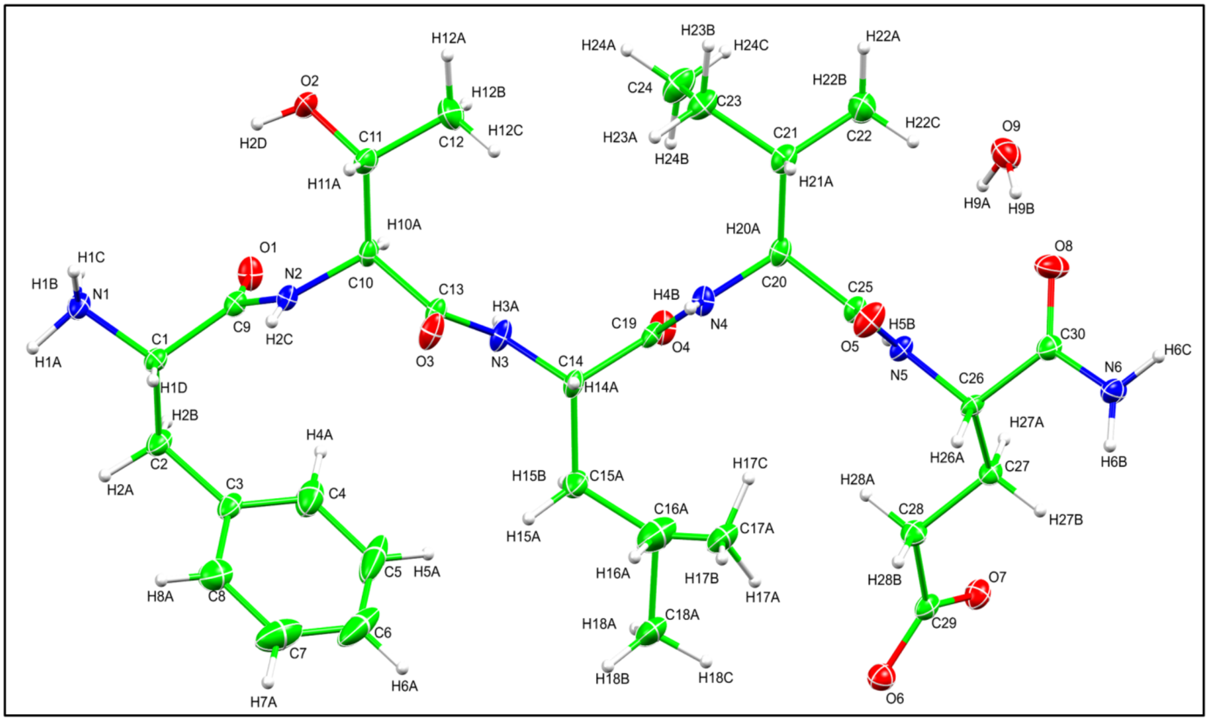

Supplementary Figure 9. View of the asymmetric unit as determined for 5-mer single-crystals by XRD. Thermal ellipsoids are shown at 50% probability level. Hydrogen atoms were refined isotropically and are given as spheres. The leucine side-chain (carbon atoms C15-C18 and the corresponding hydrogen atoms) exists in the crystals in two alternate configurations, at a ratio of approximately 1.19:1. For clarity, only the dominant configuration is shown.

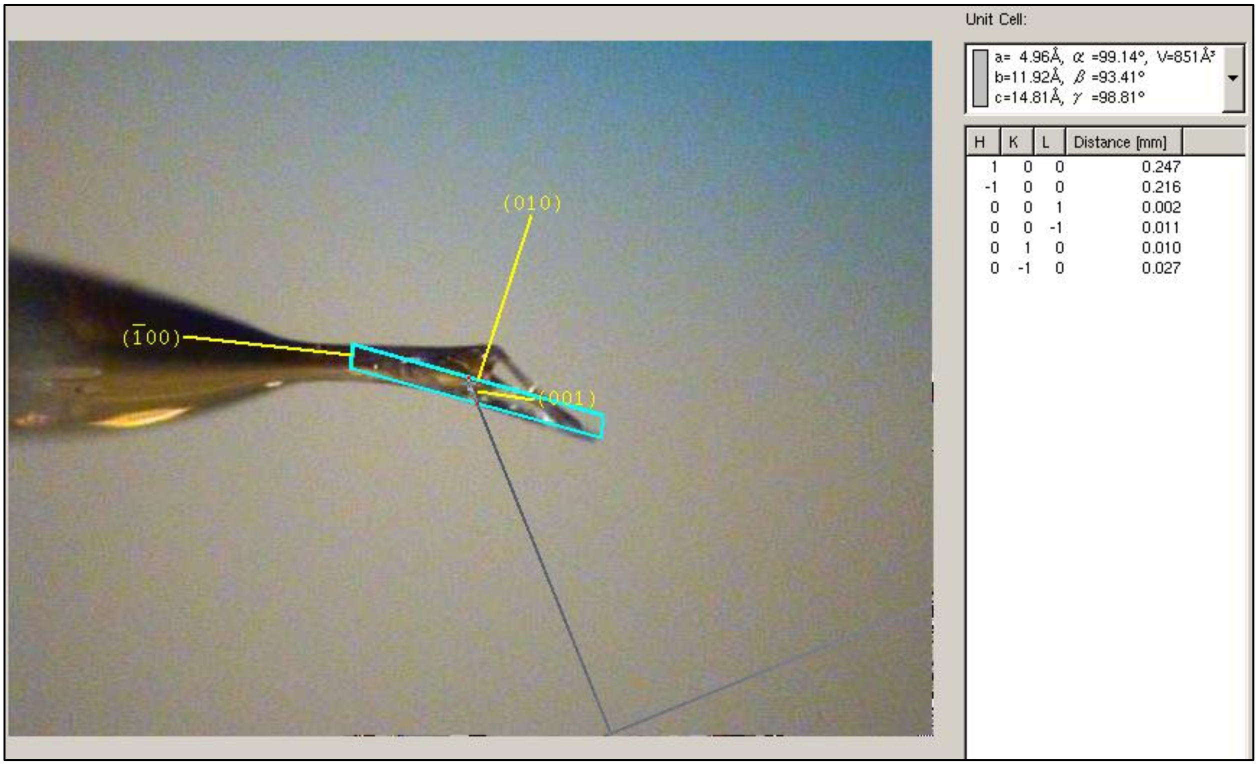

Supplementary Figure 10. Unit cell measurement of a 5-mer crystal in respect to crystal morphology. A crystal is shown mounted on a MiTeGen loop. The crystal is highlighted in cyan and unit cell planes with their respective Miller indices appear in yellow. The measured unit cell dimensions appear in the upper right corner. The morphological long axis of the crystal is aligned along the crystallographic *a* axis (100 plane) of the unit cell, as given in Figure 4 and Supplementary Table 3.

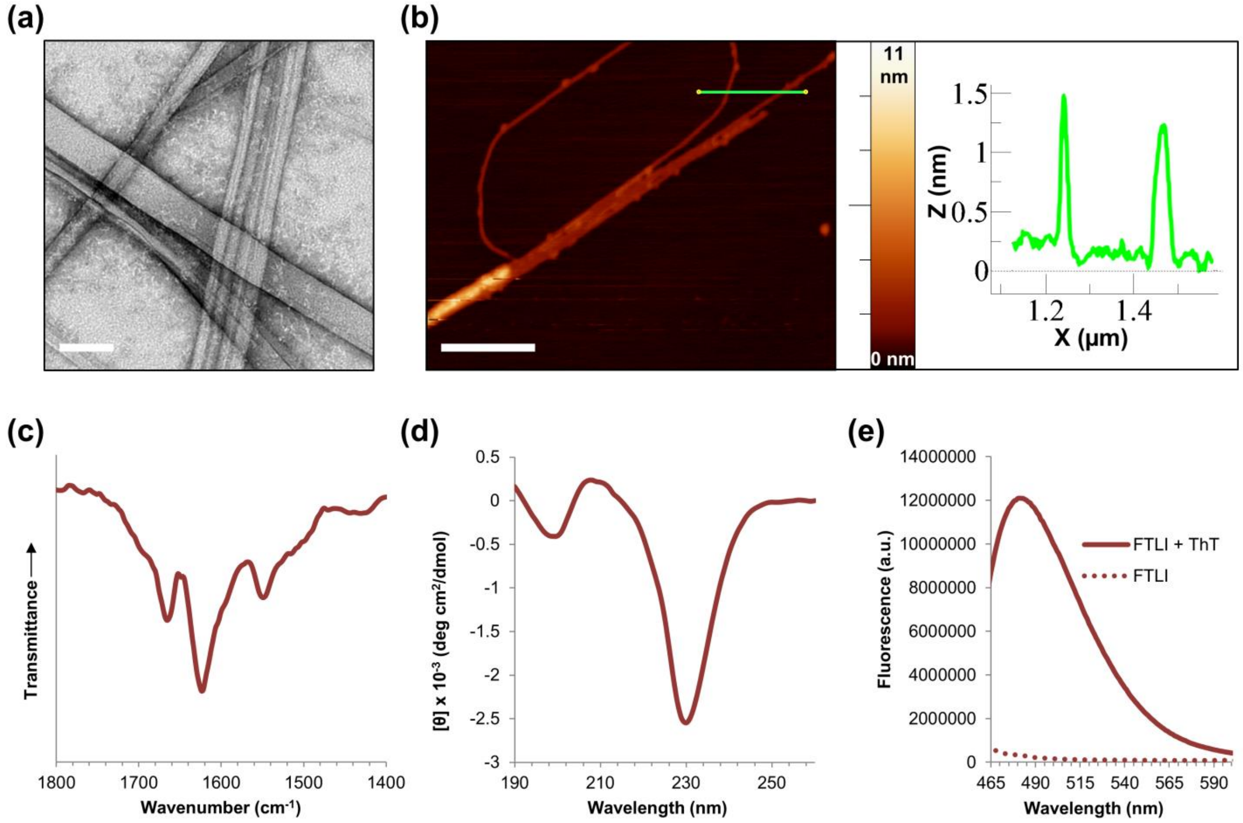

Supplementary Figure 11. Structural analysis of the self-assembling 4-mer peptide (FTLI). (**a**) TEM micrograph of the 4-mer nanofibers and nanoribbons. (**b**) AFM image of the 4-mer nanofibers; example height profile along the green line is shown on the right. (**c**) FTIR spectrum of a dried peptide sample. (**d**) CD spectrum of the peptide. (**e**) Fluorescence emission spectra of the peptide, in the presence or absence of ThT, upon excitation at 440 nm. For all assays, the peptide was prepared at 2.9 mM in phosphate buffer. Scale bars in **a, b** are 100 and 400 nm, respectively.

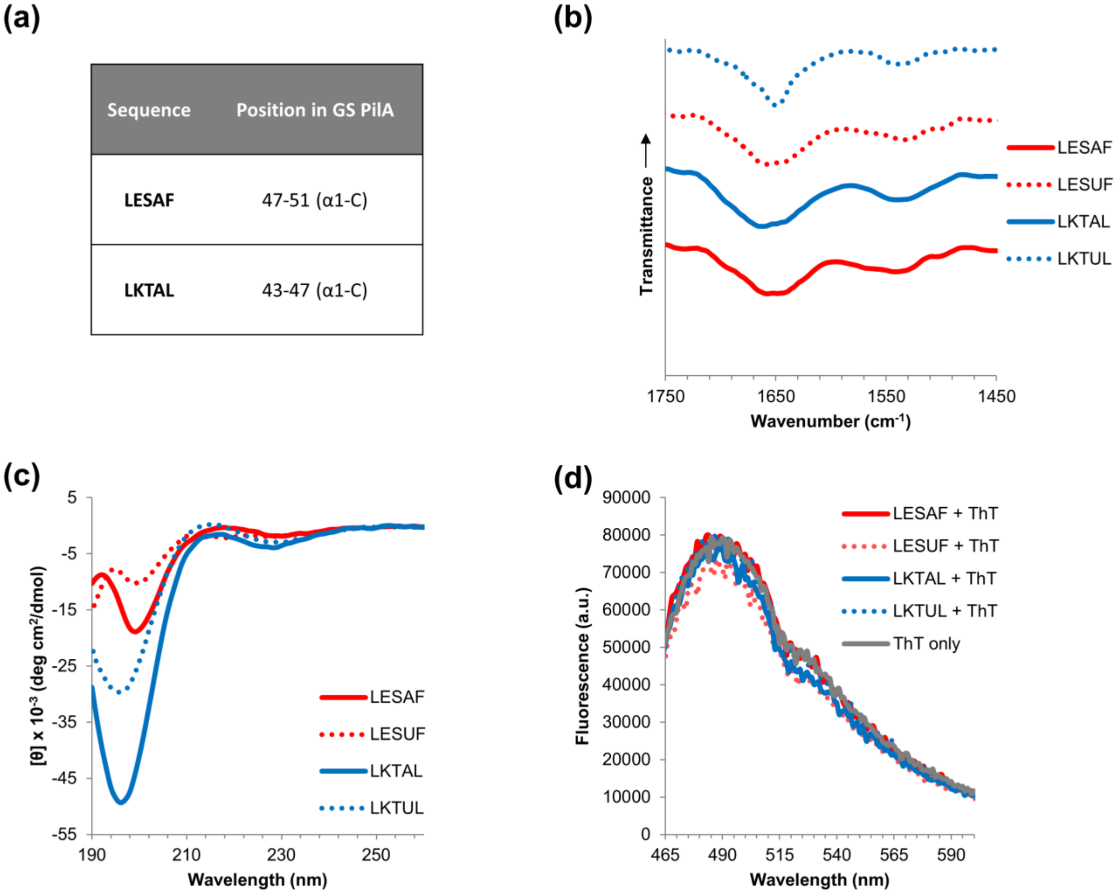

Supplementary Figure 12. Secondary structure analysis of GS PilA C-terminal non-assembling control peptides. (**a**) The investigated 5-mer peptides. Ala→Aib substituted analogues of the 5-mers were also investigated. All peptides are N-terminally acetylated and C-terminally amidated in accordance with their positions in GS PilA. (**b**) FTIR spectra of dried peptide samples. (**c**) CD spectra of the peptide solutions. (**d**) Fluorescence emission spectra of the peptides in the presence of ThT, in comparison to ThT solution with no added peptide, upon excitation at 440 nm. For all assays, peptides were prepared at 2.9 mM in phosphate buffer, except for CD spectroscopy, where the peptides were diluted with buffer to 1.45 mM. For panels **b–d**, U denotes Aib.

Supplementary Table 1. Secondary structure proportion estimation of the 20-mer peptide[a]

Method	Wavenumber (cm^{-1})	Conformation	Percentage
FTIR	1616, 1629, 1690	β-sheet	40
	1644	Random	21
	1664	3_{10} / α-helix / Random	29
	1683	β-turn	10
CD	-	β-sheet	28
	-	Random (collagen)	38
	-	α-helix	17
	-	β-turn	17

[a]The calculated relative percentages of secondary structures are the result of the quantitative analysis given in Supplementary Fig. 5.

Supplementary Table 2. Hydrogen bonding parameters from the 5-mer peptide crystal structure

	H-A [Å]	D-A [Å]	D-H-A angle	Symmetry operation
N1-H1A···O9	1.86	2.809	167	[x-1, y-1, z-1]
N1-H1B···O6	1.91	2.801	165	[x-1, y, z-1]
N1-H1B···O7	2.32	3.004	132	[x-1, y, z-1]
N1-H1C···O6	1.88	2.829	178	[x, y, z-1]
N2-H2C···O1	2.12	2.890	146	[x-1, y, z]
O2-H2D···O7	1.79	2.622	168	[x-1, y, z-1]
N3-H3A···O3	2.15	3.003	162	[x+1, y, z]
N4-H4B···O4	2.15	3.013	167	[x-1, y, z]
N5-H5B···O5	1.99	2.843	162	[x+1, y, z]
N6-H6B···O2	1.96	2.774	170	[x, y, z+1]
O9-H9A···O5	2.27	3.017	149	[x+1, y, z]
O9-H9B···O8	1.87	2.733	171	[x,y,z]

Supplementary Table 3. Crystal data and structure refinement for the 5-mer peptide crystal

	**5-mer (NH_2-FTLIE-NH_2)**
Empirical formula	C_{30} H_{50} N_6 O_9
Formula weight	638.76
Crystal system	Triclinic
Space group	*P*1
a, Å	4.9205(10)
b, Å	11.812(2)
c, Å	14.637(3)
α deg	99.369(7)
β deg	93.376(7)
γ deg	98.820(7)
V (Å^3)	826.4(3)
Z	1
d_{calc} (mg cm^{-3})	1.284
μ (mm^{-1})	0.095
Reflections	9272
Unique Reflections	3773
R_{int}	0.044
R [I>2σ (I)]	R_1 = 0.0470 wR_2 = 0.1255
Goodness of Fit	1.029

Supplementary Table 4. Conformation of sequences homologous to the investigated α1-N sequence in non-pilin proteins[a]

Homologous sequence	Conformation	Origin protein type
FTLIEL	β-strand	Ferredoxin reductase (PDB ID: 1D7Y)[1]
IELLIP	3_{10} helix/random coil	ADP-ribose-1''-monophosphatase (PDB ID: 1NJR)[2]
FTLIE	β-strand	4-Hydroxymandelate synthase (PDB ID: 3ZGJ)[3]
FTLIE	β-strand	Viral capsid protein (PDB ID: 3LQ6)[4]
FTLIE	α-helix	Hydroxynitrile lyase (PDB ID: 3WWO)[5]
FTLIE	β-strand	Receptor tyrosine kinase (PDB ID: 5AOQ)[6]
FTLIE	α-helix	Golgi reassembly stacking protein (PDB ID: 4KFV)[7]
ELLIP	α-helix	Peptide synthetase (PDB ID: 4ZXH)[8]
ELLIP	Random coil	Proteolytic neurotoxin (PDB ID: 1EPW)[9]
IELLI	α-helix	Exoribonuclease (PDB ID: 2Y35)[10]

[a]Homologous sequences were obtained by a standard sequence homology search of the α1-N sequence
FTLIELLIP against the PDB using the BLAST program[11]. Presented are the 10 highest scored non-pilin non-redundant proteins, containing only exact fragments of the query sequence.

Supplementary References

1. Senda, T. et al. Crystal structure of NADH-dependent ferredoxin reductase component in biphenyl dioxygenase. *J. Mol. Biol.* **304,** 397-410 (2000).
2. Kumaran, D., Eswaramoorthy, S., Studier, F.W. & Swaminathan, S. Structure and mechanism of ADP-ribose-1″-monophosphatase (Appr-1″-pase), a ubiquitous cellular processing enzyme. *Protein Sci.* **14,** 719-726 (2005).
3. Pratter, S.M. et al. Inversion of enantioselectivity of a mononuclear non-heme iron (II)-dependent hydroxylase by tuning the interplay of metal-center geometry and protein structure. *Angew. Chem. Int. Ed.* **52,** 9677-9681 (2013).
4. Taube S, et al. High-resolution X-ray structure and functional analysis of the murine norovirus 1 capsid protein protruding domain. *J. Virol.* **84,** 5695-5705 (2010).
5. Nakano, S., Dadashipour, M. & Asano, Y. Structural and functional analysis of hydroxynitrile lyase from *Baliospermum montanum* with crystal structure, molecular dynamics and enzyme kinetics. *Biochim. Biophys. Acta, Proteins Proteomics* **1844,** 2059-2067 (2014).
6. Jenni, S., Goyal, Y., von Grotthuss, M., Shvartsman, S.Y. & Klein, D.E. Structural basis of neurohormone perception by the receptor tyrosine kinase Torso. *Mol. Cell* **60,** 941-952 (2015).
7. Feng, Y. et al. Structural insight into Golgi membrane stacking by GRASP65 and GRASP55 proteins. *J. Biol. Chem.* **288,** 28418-28427 (2013).
8. Drake E.J. et al. Structures of two distinct conformations of holo-non-ribosomal peptide synthetases. *Nature* **529,** 235-238 (2016).
9. Swaminathan, S. & Eswaramoorthy, S. Structural analysis of the catalytic and binding sites of *Clostridium botulinum* neurotoxin B. Nat. Struct. Mol. Biol. **7,** 693-699 (2000).
10. Jinek, M., Coyle, S.M. & Doudna, J.A. Coupled 5′ nucleotide recognition and processivity in Xrn1-mediated mRNA decay. *Mol. cell* **41,** 600-608 (2011).
11. Altschul, S. F. *et al.* Gapped BLAST and PSI-BLAST: a new generation of protein database search programs. *Nucleic Acids Res.* **25,** 3389-3402 (1997).
